# Magnetic separation-based blood purification: a promising new approach for the removal of disease-causing compounds?

**DOI:** 10.1186/s12951-015-0110-8

**Published:** 2015-08-08

**Authors:** I K Herrmann, A A Schlegel, R Graf, W J Stark, Beatrice Beck-Schimmer

**Affiliations:** Institute of Anesthesiology, University Hospital Zurich, Rämistrasse 100, 8091 Zurich, Switzerland; Institute of Physiology and Zurich Center for Integrative Human Physiology, University of Zurich, Winterthurerstrasse 190, 8057 Zurich, Switzerland; Department of Surgery, Swiss HPB and Transplant Center, University Hospital Zurich, Rämistrasse 100, 8091 Zurich, Switzerland; Institute for Chemical and Bioengineering, ETH Zurich, Vladimir-Prelog-Weg 1-5/10, 8093 Zurich, Switzerland

**Keywords:** Blood purification, Intoxication, Magnetic separation, Magnetic nanoparticles, Sepsis

## Abstract

Recent studies report promising results regarding extracorporeal magnetic separation-based blood purification for the rapid and selective removal of disease-causing compounds from whole blood. High molecular weight compounds, bacteria and cells can be eliminated from blood within minutes, hence offering novel treatment strategies for the management of intoxications and blood stream infections. However, risks associated with incomplete particle separation and the biological consequences of particles entering circulation remain largely unclear. This article discusses the promising future of magnetic separation-based purification while keeping important safety considerations in mind.

## Background

**Fig. 1:**
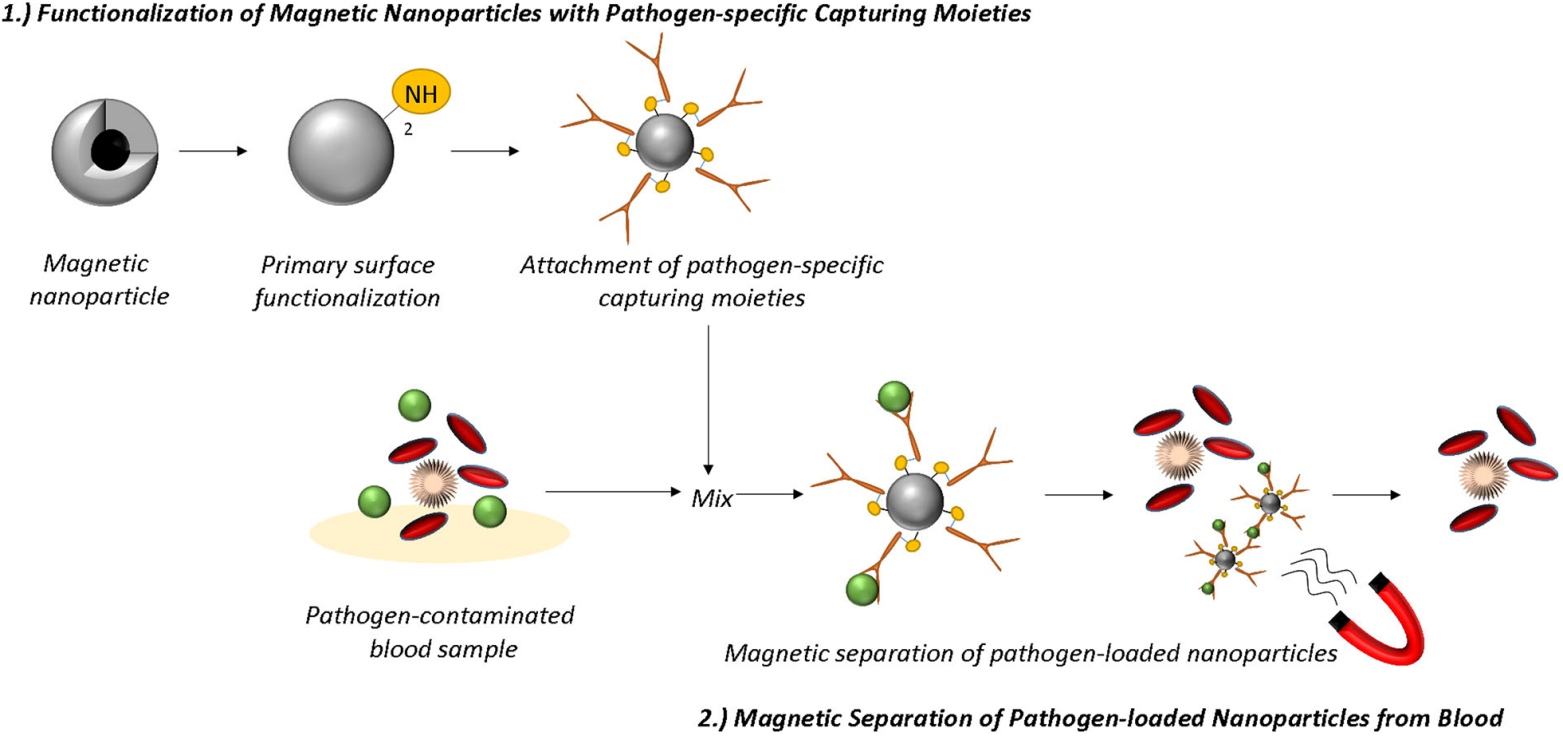
Principle of magnetic separation-based blood purification: elimination of pathogens.

The direct removal of disease-causing compounds is an inherently attractive treatment modality for a range of pathological conditions, including intoxications and blood stream infections [1]. While low molecular weight compounds (potassium, urea, etc.) are routinely removed from blood circulation by membrane-based processes, such as hemodialysis and hemofiltration [2], high molecular weight targets are only accessible by sorption-based processes e.g. hemoadsorption and hemoperfusion, where blood is pushed at high flow rates through adsorbent cartridges. In spite of promising initial findings, the practical use of hemoperfusion is still controversial and concerns have been raised due to potential side effects such as unspecific protein adsorption, loss of blood cells (e.g. platelets) and possible activation of coagulation and inflammation pathways during operation. Compared to porous membranes, the use of free-floating nano-sized particles exhibits significant benefits in terms of surface accessibility (no pore diffusion, shorter contact times), but this comes at a price: the pathogen-loaded particles need to be removed from the blood. Recently, it has been demonstrated that magnetic (nano-)particles can be employed to bind pathogenic substances on their surface, followed by a re-collection by magnetic separation. In magnetic separation-based blood purification, capturing agents attached to tiny magnetic nanoparticles are injected into an extracorporeal blood circuit (Fig. 1). They then form a complex consisting of the target compounds attached to the magnetic particle which can be rapidly removed from blood by magnetic separation. The performance of such blood cleansing processes is essentially determined by the target-ligand binding (binding site accessibility, specificity, contact time), the throughput and, most critically, the efficiency of the magnetic separation process.

## Review

**Fig. 2:**
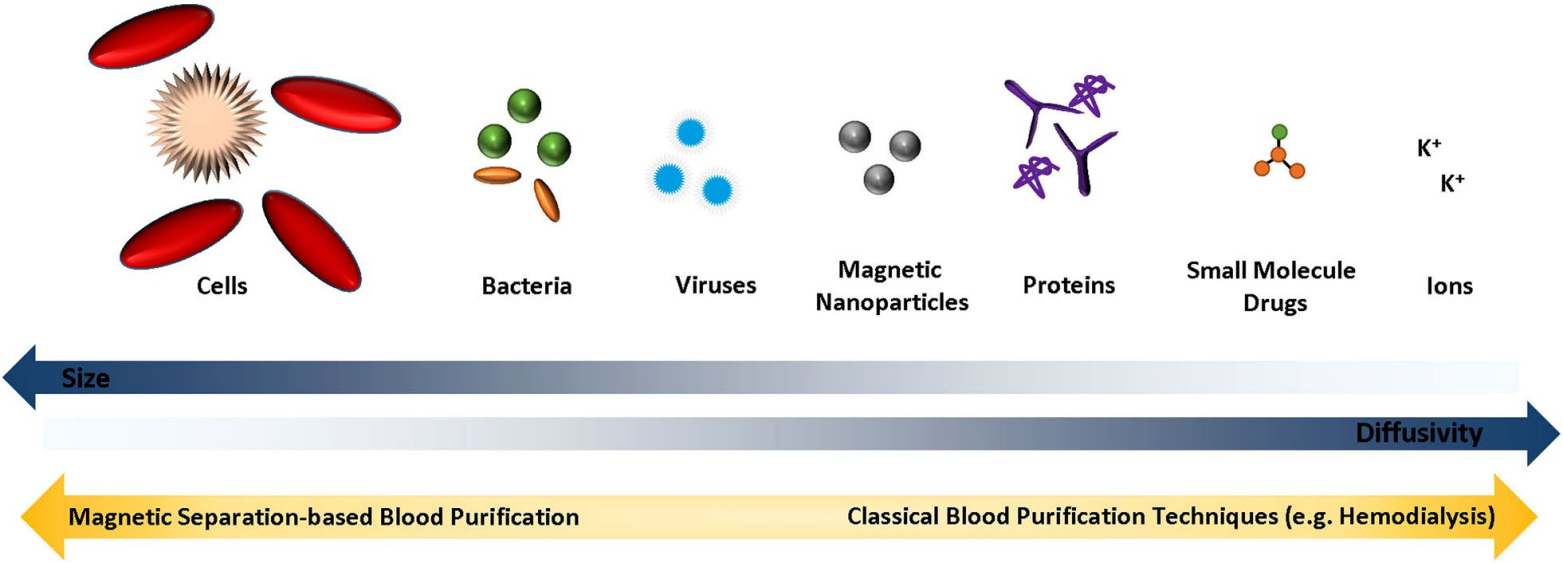
Size and diffusivity of various biologically relevant target compounds for blood purification. The larger the size of the target compound, the smaller the diffusion coefficient. Magnetic blood purification may offer a promising alternative to diffusion-based blood purification.

Magnetic separation-based blood purification is especially attractive for the removal of high molecular weight compounds, which are poorly removed by conventional (diffusion-based) blood purifications systems (e.g. dialysis, hemoadsorption) (Fig. 2) [2]. The small size, the high surface-to-volume ratio and the high mobility of nanoparticles allow short diffusion distances and hence increased binding efficiencies even for high molecular weight compounds. A range of chemically diverse target compounds, including heavy metal ions (uranyl [3], lead [4–6] and cadmium ions [7], small molecule drugs (digoxin [5, 6, 8], diazepam [9]), proteins (cytokines [5, 8]), bacteria and bacterial compounds [10] have been successfully removed ex vivo from whole blood in the past decade (Table 1). However, such targeted compound removal generally requires the use of magnetic beads with a pathogen-specific capturing agent and thus has been significantly limiting the future applicability of magnetic blood purification. Particularly, urgent medical situations, such as acute intoxications or blood stream infections where the disease-causing factor is unknown, remain challenging. In their recent study, Kang et al. [1] present a very promising capturing agent in scenarios of systemic infections that omits the necessity of first identifying the disease-causing factor. The mannose binding lectin (MBL) captures a wide range of pathogens (gram-negative, gram-positive bacteria, and fungi) and allows rapid therapeutic intervention. This is particularly relevant in sepsis patients, where identification of the causing microbe based on blood cultures typically takes 24–48 h (with a high rate of false negatives). As every hour in delayed treatment onset leads to an increased patient mortality of up to 9% [11], broad spectrum antibiotics are generally administered early. However, such overuse of antibiotics leads to antibiotic-resistant strains, increased costs and other important side effects. While magnetic capturing of circulating bacterial pathogens is very promising in the experimental setting [1], the impact on survival in human sepsis where bacterial loads in the blood are variable, and generally much lower than in animal models, remains to be investigated.

**Table 1:** Compound removed from whole blood by magnetic separation-based blood purification

Compound removed from whole blood	Model	Publication
Uranyl ions	In vitro	Wang et al. [3]
Lead ions	In vitro	Lee et al. [4] Herrmann et al. [5]
	In vivo (rat)	Herrmann et al. [6]
Cadmium ions	In vitro	Jin et al. [7]
Digoxin	In vitro	Herrmann et al. [5] Herrmann et al. [8]
	In vivo (rat)	Herrmann et al. [6]
Diazepam	In vitro	Cai et al. [9]
Interleukin-6 (IL-6)	In vitro	Herrmann et al. [5]
Interleukin-1β (IL-1β)	In vitro	Herrmann et al. [8]
Lipopolysaccharide (LPS), *Escherichia coli*	In vitro	Herrmann et al. [10]
Endotoxins, gram-negative and gram-positive bacteria, fungi	In vitro and in vivo (rat)	Kang et al. [1]

When bringing magnetic blood purification processes closer to clinical evaluation, safety of operation becomes pivotal. Extracorporeal blood purification has been suggested previously to provide a possible alternative to direct in vivo application (injection) of magnetic nanoparticles and to prevent off-target accumulation of magnetic capturing agents (e.g. in the liver or lung). Recent studies have shown that the capturing efficiency of magnetic iron oxide nanoparticles is significantly decreased under clinically desirable blood flow rates, thereby potentially compromising the procedure’s efficiency and safety [12]. Blood flow partition in front of the magnetic separator has been suggested as a valid method to decrease the perfusion flow in the magnetic separator while keeping the throughput at an acceptably high rate [1, 8]. We recently showed that ferromagnetic iron nanoparticles were retained at high efficiency under high flow rates and that magnetic bead concentration after the separator was below detection limit after a single pass [12]. However, ultra-sensitive particle detection in samples with high matrix complexity (e.g., blood, tissue) are urgently warranted as even state-of-the-art elemental analytical measurements (e.g., Inductively Coupled Plasma Mass Spectrometry) encounter significant limitations and reach detection limits in the order of >1 µg particles per gram of sample (where 1 g of particles corresponds to ~10^18^ single particles). Magnetic measurements for ultrasensitive magnetic nanoparticle detection are now increasingly being explored, which would allow detection of off-target accumulation of nanomaterial and biodegradation of nanomaterials, which in turn could initiate acute and long-term effects such as tumorigenesis, fibrosis and toxic effects.

Other important safety aspects include non-specific adsorption of blood constituents (coagulation and complement factors, cells, etc.) as well as activation of inflammatory reactions in the blood compartment [13]. Such possible side effects have to be evaluated in detail and ruled out before this new operation is translated into a clinical scenario.

Unfortunately, there is an ever growing disequilibrium between manuscripts reporting on the synthesis of new nanomaterials and their promising applications and studies actually performing comprehensive risk evaluation of the synthesized materials [14]. At present, risk analysis using relevant exposure conditions remains to be the bottle neck when translating promising nanomaterial-based approaches. Hence, it is of major importance to establish strategies to gain insight into the potential risks—both short-term and long-term—associated with magnetic blood purification and, to balance risks adequately with therapeutic benefits.

## Conclusions

In summary, extracorporeal magnetic separation-based blood purification is a promising strategy to rapidly and selectively remove high molecular weight compounds from blood. The technique has been successfully evaluated in vivo in experimental settings investigating the clinically relevant scenarios of intoxication and sepsis in rat models [1]. However, for translation, ultra-sensitive particle detection systems and risk evaluation strategies are needed in order to better understand relevant exposure scenarios and the therewith associated benefit-risk ratio. Once safety issues have been ruled out, magnetic separation-based blood purification may become an attractive treatment modality enabling rapid removal of poorly accessible high molecular weight disease-causing compounds from blood, potentially bridging the time to run diagnostic tests and establish a suitable therapy.

## Abbreviation

MBL: mannose binding lectin
